# The anti-apoptotic effect of regucalcin is mediated through multisignaling pathways

**DOI:** 10.1007/s10495-013-0859-x

**Published:** 2013-05-14

**Authors:** Masayoshi Yamaguchi

**Affiliations:** Department of Hematology and Biomedical Oncology, Emory University School of Medicine, 1365 C Clifton Road, Atlanta, GA 30322 USA

**Keywords:** Regucalcin, Apoptosis, Calcium signaling, Endonuclease, Caspase-3, Bcl-2, Nucleus

## Abstract

Regucalcin (RGN/SMP30) was originally discovered in 1978 as a calcium-binding protein that does not contain the EF-hand motif of as a calcium-binding domain. The name, regucalcin, was proposed for this calcium-binding protein, which can regulate various Ca^2+^-dependent enzymes activation in liver cells. The regucalcin gene is localized on the X chromosome, and its expression is mediated through many signaling factors. Regucalcin plays a pivotal role in regulation of intracellular calcium homeostasis in various cell types. Regucalcin also has a suppressive effect on various signaling pathways from the cytoplasm to nucleus in proliferating cells and regulates nuclear function in including deoxyribonucleic acid (DNA) and ribonucleic acid (RNA) synthesis. Overexpression of endogenous regucalcin was found to suppress apoptosis in modeled rat hepatoma cells and normal rat kidney proximal epithelial NRK52 cells induced by various signaling factors. Suppressive effect of regucalcin on apoptosis is related to inhibition of nuclear Ca^2+^-activated DNA fragmentation, Ca^2+^/calmodulin-dependent nitric oxide synthase, caspase-3, Bax, cytochrome C, protein tyrosine kinase, protein tyrosine phosphatase in the cytoplasm and nucleus. Moreover, regucalcin stimulates Bcl-2 mRNA expression and depresses enhancement of caspase-3, Apaf-1 and Akt-1 mRNAs expression. This review discusses that regucalcin plays a pivotal role in rescue of apoptotic cell death, which is mediated through various signaling factors.

## Introduction

Regucalcin was discovered in 1978 as a calcium-binding protein that does not contain the EF-hand motif of as a calcium-binding domain [1–5]. The name, regucalcin, was proposed for this calcium-binding protein, which regulates various Ca^2+^- or Ca^2+^/calmodulin-dependent enzymes activation in liver cells [4, 5]. Regucalcin (RGN) and its gene (*rgn*) are identified in over 15 species consisting of regucalcin family [6–8]. Regucalcin is highly conserved in vertebrate species throughout evolution [6]. The regucalcin gene is localized on the X chromosome and its organization consists of seven exons and six introns [9–11]. The regucalcin gene expression is enhanced through calcium and other signaling factors; AP-1, NF1-A1, RGPR-p117 and β-catenin are identified as a transcription factor that enhances transcription activity [8]. Regucalcin mRNA expression and its protein content are pronounced in the liver and kidney cortex of rats, although it is also expressed in other tissues and cell types [12–14]. The protein named as senesence marker protein-30 (SMP30), which is identical to regucalcin, was also reported after discovery of regucalcin [15, 16].

Regucalcin plays a multifunctional role in cell regulation; maintaining of intracellular calcium homeostasis in many cell types, suppressions of various signaling pathways from the cytoplasm to nucleus in proliferating cells, and regulation of nuclear functions including DNA and RNA synthesis [5, 14, 17–19]. Regucalcin has also been shown to suppress protein synthesis and activate proteases, suggesting an involvement in protein turnover in cells [20, 21]. Thus, regucalcin plays a multifunctional role in cell regulation.

There is growing evidence that overexpression of endogenous regucalcin suppresses apoptosis in modeled liver (rat hepatoma H4-II-E) cells and normal rat kidney proximal epithelial NRK52E cells that are induced through various signaling factors. Regucalcin may play an important role in rescue of apoptotic cell death. This review has been written to outline the recent advances that have been made concerning a suppressive role of regucalcin in the regulation of apoptosis and will discuss the mechanism by which regucalcin suppresses apoptosis.

## Regucalcin suppresses apoptosis in modeled liver cells

### Regucalcin inhibits apoptosis-related NO synthase activity

NO may be important as a signaling factor in many cells [22], and it plays a role in apoptosis of hepatoma cells [23]. NO mediates apoptosis by d-galactosamine in a primary culture of rat hepatocytes [24]. Regucalcin has a suppressive effect on Ca^2+^/calmodulin-dependent NO synthase activity in the cloned rat hepatoma H4-II-E cells [25], suggesting its role in apoptosis [25]. Suppressive effect of regucalcin on NO synthase activity was also seen in presence of trifluoperazin (TFP), an inhibitor of calmodulin, or ethyleneglycol bis (2-amino-ethylether)-*N*, *N*, *N*′, *N*′-tetraacetic acid (EGTA), a chelator of Ca^2+^ [25]. Regucalcin may have a suppressive effect on NO synthase activity due to binding calmodulin and/or enzyme independently on Ca^2+^ in proliferating cells.

The role of endogenous regucalcin in cell regulation has been shown in regucalcin/pCXN2-transfected hepatoma H4-II-E cells that overexpress regucalcin stably [26]. The regucalcin content of regucalcin/pCXN2-transfected cells used in this study was 19.7-fold as compared with that of the parental wild-type H4-II-E cells and pCXN2 vector-transfected cells (mock type) [26]. Overexpressing of endogenous regucalcin has also shown to have a suppressive effect on NO synthase activity in H4-II-E cells (transfectants) [25]. This decrease was completely abolished in presence of anti-regucalcin monoclonal antibody in the reaction mixture [25]. Moreover, the effect of Ca^2+^/calmodulin addition in increasing NO synthase activity in H4-II-E cells (wild-type) was also depressed in transfectants [25]. Endogenous regucalcin may have a suppressive effect on Ca^2+^/calmodulin-dependent NO synthase activity in H4-II-E cells. Moreover, NO synthase activity was enhanced in H4-II-E cells cultured with 10 % FBS as compared with that of 1 % FBS [25]. Increase of NO synthase activity in H4-II-E cells cultured with 10 % FBS was enhanced in presence of anti-regucalcin monoclonal antibody [25], supporting the view that endogenous regucalcin reveals a suppressive effect on enhancement of NO synthase activity in proliferating cells.

A high concentration of NO, which is produced from inducible NO synthase, has been shown to suppress cell proliferation [27] and induce cell apoptosis [28]. It has been reported that a low concentration of NO, which is produced by endothelial NO synthase, protects against cytotoxic effects of reaction oxygen species in cells [29]. Whether endogenous regucalcin suppresses NO production in H4-II-E cells is unknown at present, although it inhibites NO synthase activity [25]. It is speculated, however, that regucalcin depresses NO production in H4-II-E cells. Endogenous regucalcin may have the anti-apoptotic effect due to suppressing NO production in hepatoma cells.

### Regucalcin suppresses tumor necrosis factor α (TNF-α)-induced apoptosis

TNF-α and NO mediate apoptosis induced by d-galctosamine in a primary culture of rat hepatocytes [27, 28]. TNF-α induces apoptosis in mammary adenocarcinoma cells by an increase in intranuclear free Ca^2+^ concentration and DNA fragmentation [27]. When subconfluent H4-II-E cells were cultured in a medium without FBS in the presence of TNF-α, TNF-α (0.1–10 ng/ml) caused a significant decrease in the number of H4-II-E cells (wild-type), inducing cell death. Overexpressing of regucalcin in H4-II-E cells (transfectants) has been found to prevent the effect of TNF-α in decreasing cell number [30]. Thus, overexpressing of regucalcin has a rescue effect on cell death induced with the higher concentration of TNF-α (10 ng/ml), supporting the view that regucalcin has a suppressive effect on TNF-α-induced cell death [30].

Culture with *N*ω-nitro-l-arginine methylester (NAME), an inhibitor of NO synthase, has been shown to have a suppressive effect on TNF-α-induced cell death [30]. Regucalcin inhibited Ca^2+^/calmodulin-dependent NO synthase activity in H4-II-E cells [30]. Suppressive effect of regucalcin on cell death may be partly resulted from the depression of NO production enhanced after TNF-α stimulation in H4-II-E cells. The effect of caspase inhibitor on TNF-α-mediated cell death in H4-II-E cells has also been shown [30]. TNF-α-induced cell death was depressed after culture with caspase inhibitor in wild-type cells and transfectants [30], suggesting that TNF-α-induced cell death is partly involved activation of caspases in H4-II-E cells. Regucalcin may have a suppressive effect on activation of caspases in the cells.

Moreover, overexpression of regucalcin has been shown to have a suppressive effect on TNF-α-induced apoptosis in human hepatoma HepG2 cells [31]. Akt, which is a survival factor in cells, has been shown to activate in transfectants [31].

Interestingly, the effect of TNF-α in inducing apoptosis has been shown to enhance in the hepatocytes obtained from regucalcin-deficiency mice in vivo [32]. Moreover, this animal was found to enhance liver injury after treatment with anti-Fas antibody [32]. Regucalcin may play a protective role on apoptosis in vivo.

### Regucalcin suppresses lipopolysacharide (LPS)-induced apoptosis

LPS induces cell apoptosis [33, 34]. LPS causes a decrease in the number of H4-II-E cells (wild-type), inducing cell death and apoptosis [35]. This decrease was completely protected by overexpressing of endogenous regucalcin with culture for 12–48 h [35]. Thus, overexpression of endogenous regucalcin has suppressive effects on LPS-stimulated cell death and apoptosis.

LPS modulates the expression of a large number of genes that favor apoptosis of fibroblastic cells, which are dependent upon activation of caspase-8 [33]. There is evidence that LPS-induced cell death is mediated through accumulation of reactive oxygen species and activation of p38 in rat brain cortex and hippocampus [33]. Culture with LPS caused a significant decrease in Ca^2+^/calmodulin-dependent NO synthase activity in H4-II-E (wild-type) cells [35]. LPS-induced decrease in NO synthase activity was preventeed in transfectants overexpressing regucalcin [35]. LPS-induced cell death may be not resulted from NO production in hepatoma cells, and suppressive effect of regucalcin on LPS-induced cell death may be not involved in NO in the cells. Moreover, LPS-induced cell death was protected after culture with caspase-3 inhibitor [35]. Depressive effect of regucalcin on LPS-induced cell death may be partly related to its inhibitory effect on caspase-3 in hepatoma cells.

### Regucalcin suppresses various signaling inhibitors-induced apoptosis

An induction of apoptosis is partly mediated through pathway of protein kinase. The death of H4-II-E cells (wild-type) has been found to be induced after culture with PD 98059, a ERK inhibitor, dibucaine, an inhibitor of Ca^2+^-dependent protein kinase, or staurosporine, a potent inhibitor of protein serine/threonin kinases (protein kinase C), suggesting that various inhibitors-induced cell death is partly involved in inhibition of protein kinases [35]. Overexpression of regucalcin rescued death of H4-II-E cells cultured with PD 98059 or dibucaine [35]. This effect was not observed after culture with staurosporine. PD 98059 induces apoptosis that is mediated through inactivation of Bcl-2 due to increasing in phosphorylated Bcl-2 in human prostate cancer cells [36]. Dibucaine has been shown to activate various caspases, such as caspase-3, -6, -8, and -9 (-like) activities, but not caspase-1 (-like) activity, and induce mitochondrial membrane depolarization and release of cytochrome C from mitochondria into the cytosol in leukemia cells (HL-60) [37]. Staurosporine induces apoptosis in Chang liver cells through a mitochondria-caspase-dependent pathway, which is closely correlated to a decrease in Bcl-2 and Bcl-XL levels in cancer cells [38]. Regucalcin may partly suppress inactivation of Bcl-2 or activation of caspases that are the mechanism by which PD 98059 or dibucaine induces apoptosis [38].

### Regucalcin suppresses Ca^2+^-stimulated DNA fragmentation

Apotosis is evoked by nuclear DNA fragmentation that is mediated through activation of endonuclease. Isolated rat liver nucleus contains a DNA endonuclease activity dependent upon Ca^2+^, and Ca^2+^ results in extensive DNA hydrolysis [39]. Ca^2+^ dependence of DNA fragmentation process is based on increased DNA endonuclease activity dependent upon sub-micromolar Ca^2+^ when the nucleus is reconstituted with NAD^+^ and ATP [39]. This endonuclease activity may be responsible for DNA fragmentation occurring during programmed cell death (apoptosis) and certain forms of chemically induced cell killing [39, 40].

Regucalcin has been found to have a suppressive effect on Ca^2+^-activated DNA fragmentation in isolated rat liver nuclei [41]. Among various metals, Ca^2+^ was shown to uniquely stimulate in vitro DNA fragmentation in isolated rat liver nuclei [41]. This increase was seen after addition of 1.0 μM Ca^2+^, in agreement with previous work [39, 41]. Presence of regucalcin (0.5-2.0 μM) in reaction mixture evoked a complete suppression of activation of liver nuclear DNA fragmentation, when 10 μM Ca^2+^ was added into the reaction mixture. This inhibition was not seen in the presence of Ca^2+^ at 25 or 50 μM. Thus, regucalcin has been shown to have an inhibitory effect on DNA fragmentation with a comparatively lower concentration of Ca^2+^ (5.0 and 10 μM*)* [41]. Suppressive effect of regucalcin on nuclear DNA fragmentation may be partly based on binding of Ca^2+^.

DNA fragmentation in rat liver nucleus has been reported to be stimulated through Ca^2+^-calmodulin [39], which exists in the nuclei [42]. Addition of calmodulin (10 and 20 μg/ml) in the reaction mixture did not enhance nuclear Ca^2+^ (10 μM)-activated DNA fragmentation [41]; however, nuclear endogenous calmodulin may enhance Ca^2+^-activated DNA fragmentation. Regucalcin suppresses nuclear DNA fragmentation in the presence or absence of exogenous calmodulin and Ca^2+^ [41]. Regucalcin may directly suppress endonucleas activity in the liver nuclei.

Ca^2+^ plays an important role in the regulation of nuclear functions [43, 44]. A sustained increase in cytosolic Ca^2+^ level precedes the activation of DNA fragmentation that is characteristic of programmed cell death (apoptosis) and in certain forms of chemically induced cell killing [39, 40]. Finding, that regucalcin depresses activation of nuclear Ca^2+^-induced DNA fragmentation, suggests a role of regucalcin in regulation of apotosis.

### Regucalcin suppresses on calcium signaling-induced apoptosis

Calcium channel blockers, the endoplasmic reticulum Ca^2+^-ATPase inghibitor thapsigargin and calcium ionophores are potent to lead several cell types to apoptosis [45, 46]. Thapasigargin is an inhibitor of Ca^2+^-ATPase in the endoplasmic reticulum (Ca^2+^ store) in cells, and treatment with thapsigargin causes an elevation of sustained Ca^2+^ concentration in cells and induces apoptosis in hepatoma cells [47]. Experiments on the nucleus isolated from cells clearly demonstrate the induction of Ca^2+^-dependent endonuclease activity during triggering apoptosis events [47]. Rises in intracellular Ca^2+^ concentration activates endonuclease that mediates DNA cleavages into oligonucleosome fragments [48]. Regucalcin has been shown to have an inhibitory effect on Ca^2+^-activated DNA fragmentation in isolated rat liver nucleus [41].

Thapsigargin-induced DNA fragmentation in the hepatoma cells is not altered after culture with caspase inhibitor, suggesting that thapsigargin-mediated apoptosis is independent on activation of caspases [48]. Overexpression of regucalcin in hepatoma cells suppresses thapsigargin-induced DNA fragmentation [36]. This effect is not further enhanced after culture with caspase inhibitor [36]. Presumably, regucalcin has a suppressive effect on thapsigargin-mediated cell death due to protecting rise in intracellular Ca^2+^ concentration in hepatoma cells. Regucalcin has been shown to maintain intracellular Ca^2+^ homeostasis due to activating Ca^2+^ pum enzymes in the plasma membranes, mitochondria, and endoplasmic reticulum of rat liver cells [17, 18].

Calcium entry into cells induces cell death [36, 49]. Culture with Bay K 8644, an antagonist of Ca^2+^ entry in cells, caused a significant increase in the death of hepatoma H4-II-E cells (wild-type) [41]. Culture with Bay K 8644 did not cause cell death of H4-II-E cells overexpressing regucalcin [41]. Overexpression of regucalcin in H4-II-E cells suppresses DNA fragmentation enhanced by Bay K 8644. Regucalcin has a suppressive effect on Ca^2+^ entry-mediated cell death due to depressing rise in intracellular Ca^2+^ concentration in hepatoma cells. In addition, regucalcin may suppresse effect of Ca^2+^ on DNA fragmentation in the nucleus of H4-II-E cells.

### Regucalcin suppresses insulin or insulin growth factor-I (IGF-I)-induced apoptosis

The effect of insulin or IGF-I on cell death and apoptosis in H4-II-E cells has been not known. H4-II-E cells were cultured in a medium containing, either vehicle, insulin, IGF-I, epinephrine, or transforming growth factor-β (TGF-β) in absence of FBS [50]. The number of wild-type cells was decreased after culture of insulin or IGF-I [50]. Agarose gel electrophoresis showed presence of low-molecular-weight DNA fragments of adherent wild-type cells cultured in the presene of insulin or IGF-I [36]. The effect of insulin or IGF-I in stimulating cell death and DNA fragmentation H4-II-E cells was suppressed by overexpression of regucalcin [50].

The effect of insulin in decreasing the number of H4-II-E cells is protected in presence of caspase-3 inhibitor [50]. The effect of IGF-I on cell death, however, is observed in presence of caspase-3 inhibitor [50]. These observations suggest that the effect of insulin on cell death is involved in activation of caspase-3 and that effect of IGF-I is not dependent on caspase-3 in H4-II-E cells. The effect of IGF-I in inducing cell death in presence of caspase-3 inhibitor was completely suppressed by overexpression of regucalcin [50]. Regucalcin may depress signaling pathway of IGF-I-induced cell death, which is not mediated through caspase-3 in H4-II-E cells.

The effect of insulin or IGF-I in inducing cell death and apoptosis of H4-II-E cells is depressed in presence of N-nitro-l-arginine methylester (NAME), an inhibitor of NO synthase [50], suggesting that insulin- or IGF-induced cell death is partly involved in production of NO in H4-II-E cells. Overexpression of regucalcin has been shown to have a suppressive effect on activation of Ca^2+^/calmodulin-dependent NO synthase in H4-II-E cells [31].

The effect of IGF-I in inducing apoptosis of H4-II-E cells has been shown to reveal in presence of Bay K 8644 [50]. This effect is not seen in the case of insulin [50]. The mode of IGF-I action differs from that of insulin. It is assumed that insulin induces cell death, which is partly mediated through intracellular Ca^2+^-dependent signaling pathway in H4-II-E cells, and that IGF-I may be not mediathed through Ca^2+^-dependent signaling pathway in H4-II-E cells. The effect of IGF-I in inducing cell death in presence of Bay K 8644 was suppressed by overexpression of regucalcin [50].

Genistein has an inhibitory effect on protein tyrosine kinases and produces cell cycle arrest and apoptosis in leukemic cells [51]. Genistein was found to induce cell death of H4-II-E cells, and such effect was not seen by overexpression of regucalcin [51]. Genistein-induced cell death is partly mediated through inhibition of protein tyrosine kinase in H4-II-E cells. Regucalcin has an inhibitory effect on protein tyrosine kinase activity in the cytoplasm and nucleus of rat liver [52].

The effect of insulin in inducing cell death of H4-II-E cells was suppressed in presence of genistein [50], although this effect was not seen in case of IGF-I. The effect of IGF-I on cell death in presence of genistein was protected in the transfectants overexpressing regucalcin [50]. Regucalcin has a suppressive effect on cell apoptosis that is mediated through signaling pathways with dependent or independent on protein tyrosine kinase.

Vanadate is an inhibitor of protein tyrosine phosphatase in cells [53]. Regucalcin has been shown to have an inhibitory effect on protein tyrosine phosphatase activity in the cytoplasm and nucleus of rat liver [54]. Vanadate induced apoptosis of H4-II-E cells [50], suggesting that cell death is not involved in mechanism that is mediated through inhibition of protein tyrosine phosphatase activity. Vanadate induced cell death of transfectants overexpressing regucalcin [50], suggesting that suppressive effect of regucalcin on cell death of H4-II-E cells is independent on protein phosphatase. IGF-I stimulated cell death of H4-II-E cells overexpressing regucalcin in presence of vanadate [50], suggesting that effect of IGF-I is not mediated through protein tyrosine phosphatase in transfectants.

Thus, the effect of insulin in inducing apoptosis may be partly mediated through signaling pathway which is involved in caspase-3, Ca^2+^, NO, protein tyrosine kinase, or protein tyrosine phosphatase in H4-II-E cells. The effect of IGF-I on apoptosis of H4-II-E cells may be mediated through NO and other molecules. Overexpression of regucalcin may have a suppressive effect on signaling pathways which insulin or IGF-I induces cell death of H4-II-E cells.

### Regucalcin suppresses sulforaphane-induced apoptosis

Sulforaphane is an isothiocyanate that is present naturally in widely consumed vegetables and has a particularly high concentration in broccoli. This compound has been shown to block the formation of tumors initiated by chemicals in the rat [55]. Sulforaphane induces a cell cycle arrest, followed by cell death in HT29 human colon cancer cells [55]. Sulforaphane increases expression of the pro-apoptotic protein Bax, the release of cytochrome C from the mitochondria to the cytosol, and proteolytic cleavage of poly (ADP-ribose) polymerase in HT29 human colon cancer cells [55]. In human T cell leukemia, sulforaphane induces apoptosis due to increasing p53 and Bax protein expressions and slightly affecting Bcl-2 expression [56]. In cultured PC-3 human prostate cancer cells, sulforaphane-induced apoptosis is associated with up-regulation of Bax, down-regulation of Bcl-2 and activation of caspase-3, -9, and -8 [57]. Sulforaphane induced cell death and apoptosis in H4-II-E cells [58]. The effect of sulforaphane on apoptosis was depressed caspase-3 inhibitor, while it was not inhibited by NAME, an inhibitor of NO synthase, in H4-II-E cells [58]. Sulforaphane-induced cell death and apoptosis partly result from activation of caspase-3 in hepatoma cells. Overexpression of regucalcin was found to have suppressive effects on cell death and apoptosis induced by sulforaphane in H4-II-E cells [58]. This effect of regucalcin may be partly involved in the molecules of Bax, cytochrome C, caspase, and Bcl-2. In addition, regucalcin may have an inhibitory effect on NO synthase and Ca^2+^-dependent endonuclease activities in H4-II-E cells [24, 31].

As described above, regucalcin has been shown to have suppressive effects on cell death and apoptosis in H4-II-E cells, which are mediated through various signaling factors [36, 41, 50]. Regucalcin may have a suppressive effect on various signaling pathways that mediate apoptotic cell death. Overexpression of regucalcin has suppressive effects on cell death and apoptosis induced by TNF-α, LPS, thapsigargin, Bay K 8644, dibucaine, or PD98059, an inhibitor of protein thyrosine kinase, insulin, IGF-I, or sulforaphane in H4-II-E cells [36, 41, 50]. Signaling mechanisms that TNF-α, LPS, or other factors mediate cell death and apoptosis may be different. Suppressive effect of regucalcin on apoptotic cell death is related to its inhibitory effect on the activities of various protein kinases, NO synthase, caspase-3, or Ca^2+^-dependent endonuclease, and its activatory effect on Bcl-2. Regucalcin has suppressive effects on various signaling-mediated cell death and apoptosis and suppresses cell death and apoptosis mediated through various different signaling pathways in H4-II-E cells.

## Regucalcin rescues apoptosis in normal kidney cells

Regucalcin has been shown to express in the cloned normal rat kidney proximal tubular epithelial NRK52E cells and its expression is increased after hormonal stimulation [59]. The nuclear localization of regucalcin is enhanced after hormone stimulation in NRK52E cells [60]. NRK52E cells (transfectants) overexpressing endogenous regucalcin have been generated. Regucalcin content in this transfectants showed about 21-fold as compared with that of the parental wild-type cells. Enhancement of cell proliferation was suppressed in the transfectants overexpressing regucalcin [61]. The number of wild-type cells was decreased after culture for 42–72 h in presence of TNF-α, TGF-β, LPS, Bay K 8644, or thapsigargin [62, 63]. This effect was not seen in the transfectants overexpressing regucalcin. DNA fragmentation induced after culture with LPS, Bay K 8644, or thapsigargin were protected in the transfectants overexpressing regucalcin [62]. Thus, overexpression of regucalcin has been found to have a suppressive effect on apoptotic cell death induced by TNF-α, TGF-β, LPS, Bay K 8644, or thapsigargin in NRK52E cells. The effect of regucalcin in suppressing apoptotic cell death may be mediated through its action on various signaling pathways in NRK52E cells.

Overexpreesion of regucalcin has been found to enhance the gene expressions of NF-κB or Smad2, which is signaling factor of TNF-α or TGF-β, in NRK52E cells [63]. However, stimulatory effect of TNF-α or TGF-β on Smad2 and NF-κB mRNA expressions was not significantly enhanced in transfectants [63]. This suggests that suppressive effects of regucalcin on TNF-α- or TGF-β-induced apoptosis may not be based on NF-κB and Smad2 mRNA expressions. Suppressive effect of regucalcin on apoptosis may be related to its action on other signaling pathways.

Bcl-2 is a suppressor in apoptotic cell death [64]. Apaf-1 participates in activation of caspase-3 [65]. Akt-1 regulates survival-signaling pathways in cell death [66]. Overexpression of regucalcin caused a remarkable elevation of Bcl-2 mRNA expression in NRK52E cells, and it slightly stimulated Akt-1 mRNA expression in the cells. Apaf-1, caspase-3, or G3PDH mRNA expressions were not significantly altered in transfectants [67]. Presumably, the enhancement of Bcl-2 mRNA expression contributes to rescue of apoptotic cell death in NRK52E cells overexpressing regucalcin. Regucalcin may play a role in regulation of Bcl-2 gene expression in NRK52E cells.

TNF-α enhanced expression of caspase-3 mRNA in NRK52E cells [62]. This effect was depressed in transfectants [62], suggesting that the mechanism by which regucalcin suppresses TNF-α-induced cell death is partly related to suppression in caspase-3 mRNA expression in transfectants.

Culture with LPS caused a significant decrease in Bcl-2 mRNA expression in NRK52E cells, suggesting that this decrease is partly related to LPS-induced cell death [62]. Enhancement of Bcl-2 mRNA expression caused by overexpression of regucalcin was also seen in presence of LPS [62]. LPS-stimulated expression of Apaf-1 mRNA was suppressed after overexpression of regucalcin [62]. This may partly involve in suppression of LPS-induced cell death in NRK52E cells overexpressing regucalcin.

Culture with Bay K 8644 or thapsigargin has been shown to cause an increase in caspase-3 mRNA expression in wild-type cells, indicating that increased gene expression partly contributes to inducing apoptotic cell death [62]. This enhancement was completely depressed in transfectants. Regucalcin may have a suppressive effect on caspase-3 mRNA expression enhanced after culture with Bay K 8644 or thapsigargin in NRK52E cells. Thus, regucalcin regulates expression of Bcl-2, caspase-3, and Akt-1 mRNAs in NRK52E cells. Change in these proteins level, however, remains to be elucidated.

Overexpression of regucalcin has a suppressive effect on apoptotic cell death induced by various factors (including TNF-α, TGF-β, LPS, Bay K 8644, or thapsigargin) in NRK52E cells. Toxic factors have been reported to induce renal failure due to stimulating apoptotic cell death [67]. Regucalcin may play a role as a suppressor in inducing of apoptotic cell death in kidney proximal tubular epithelial cells.

## Prospect

**Fig. 1:**
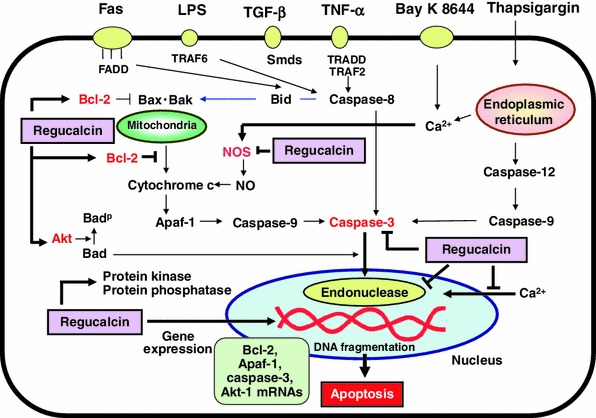
Regucalcin plays a role as suppressor in cell death and apoptosis induced through various signaling factors (including TNF-α, TGF-β, insulin, IGF-I, LPS, PD98059, dibucaine, thapsigargin, Bay K 8644, or sulforaphane). Protective effect of regucalcin on cell death and apoptosis is mediated through suppressive effects on the activities of NO synthase, protein kinase, protein phosphatase, caspase-3, or Ca^2+^-dependent and independent endonuclease and stimulatory effects on the expressions of Bcl-2 and Akt-1 mRNAs in normal cells and cancer cells

Overexpression of regucalcin rescues cell death and apoptosis induced with various factors (including TNF-α, TGF-β, LPS, insulin, IGF-I, Bay K 8644, PD98059, dibucaine, thapsigargin, or sulphoraphan), which those signaling mechanisms are different in the hepatoma cells and normal kidney cells. The anti-apoptotic effect of regucalcin is mediated through suppressive effects on various signaling pathways that mediate apoptotic cell death as summarized in Fig. 1. This effect of regucalcin is based on inhibitory effects on the activities of NO synthase, protein kinase, protein phosphatase, caspase-3, or Ca^2+^-dependent endonuclease and stimulatory effects on Bcl-2 and Akt-1 mRNA expressions. Caspase-3, which is final stage of apoptosis-inducing signaling pathways, activates nuclear endonuclease. This enzyme plays a pivotal role in nuclear DNA fragmentation. Moreover, regucalcin, which is translocated into the nucleus, has been shown to directly suppress endonuclease. This suppression may play a pivotal role as molecular mechanism by which regucalcin rescues apoptotic cell daeth that is mediated through various signaling factors. Regucalcin has been shown to suppress cell proliferation [68]. The anti-apoptotic effect of regucalcin may not be related to suppression of cell proliferation. Regucalcin may be important as a molecule to protect cells from apoptosis. Although physiological significance of the anti-apoptotic effect of regucalcin remains to be elucidated, regucalcin may play a pivotal role as a regulatory protein in signaling systems in maintaining of cell homeostasis. Targeting of regucalcin molecule may be an important in protection of cell death that is evoked in various pathophysiologica states.

## References

[1] Yamaguchi M, Yamamoto T (1978). Purification of calcium binding substance from soluble fraction of normal rat liver. Chem Pharm Bull.

[2] Yamaguchi M, Sugii K (1981). Properties of calcium-binding protein isolated from the soluble fraction of normal rat liver. Chem Pharm Bull.

[3] Yamaguchi M (1988). Physicochemical properties of calcium-binding protein isolated from rat liver cytosol: Ca^2+^-induced conformational changes. Chem Pharm Bull.

[4] Yamaguchi M, Mori S (1988). Effect of Ca^2+^ and Zn^2+^ on 5′-nucleotidase activity in rat liver plasma membranes: hepatic calcium-binding protein (regucalcin) reverses the Ca^2+^ effect. Chem Pharm Bull.

[5] Yamaguchi M, Kohama K (1992). A novel Ca^2+^-binding protein regucalcin and calcium inhibition. Regulatory role in liver cell function. Calcium inhibition.

[6] Shimokawa N, Yamaguchi M (1993). Molecular cloning and sequencing of the cDNA coding for a calcium-binding protein regucalcin from rat liver. FEBS Lett.

[7] Misawa H, Yamaguchi M (2000). The gene of Ca^2+^-binding protein regucalcin is highly conserved in vertebrate species. Int J Mol Med.

[8] Yamaguchi M (2011). The transcriptional regulation of regucalcin gene expression. Mol Cell Biochem.

[9] Shimokawa N, Matsuda Y, Yamaguchi M (1995). Genomic cloning and chromosomal assignment of rat regucalcin gene. Mol Cell Biochem.

[10] Thiselton DL, McDowall J, Brandau O, Ramser J, d’Esposito F, Bhattacharga SS, Ross MT, Hardcastle AJ, Meindl A (2002). An integrated, functionally annotated gene map of the DXS8026-ELK1 internal on human Xp11.3-Xp11.23: potential hotspot for neurogenetic disorders. Genomics.

[11] Yamaguchi M, Makino R, Shimokawa N (1996). The 5′ end seguences and exon organization in rat regucalcin gene. Mol Cell Biochem.

[12] Shimokawa N, Yamaguchi M (1992). Calcium administration stimulates the expression of calcium-binding protein regucalcin mRNA in rat liver. FEBS Lett.

[13] Yamaguchi M, Isogai M, Kato S, Mori S (1991). Immunohistochemical demonstration of calcium-binding protein regucalcin in the tissues of rats: the protein localizes in liver and brain. Chem Pharm Bell.

[14] Marques R, Maia CJ, Vaz C, Correia S, Socorro S (2013) The diverse roles of calcium-binding protein regucalcin in cell biology: from tissue expression and signalling to disease. Cell Mol Life Sci. doi:10.1007/s00018-013-1323-3 10.1007/s00018-013-1323-3 PMC11113322 23519827

[15] Fujita T, Uchida K, Maruyama N (1992). Purification of senescence marker protein-30 (SMP30) and its androgen-independent decrease with age in the rat liver. Biochim Biophys Acta.

[16] Fujita T, Shirasawa T, Uchida K, Maruyama N (1992). Isolation of cDNA clone encoding rat senescence marker protein-30 (SMP30) and its tissue distribution. Biochim Biophys Acta.

[17] Yamaguchi M (2005). Role of regucalcin in maintaining cell homeostasis and function (review). Int J Mol Med.

[18] Yamaguchi M (2011). Regucalcin and cell regulation: role as a supressor in cell signaling. Mol Cell Biochem.

[19] Laurentino SS, Correia S, Cavaco JE, Oliveira PF, de Sousa M, Barros A, Socorro S (2012). Regucalcin, a calcium-binding protein with a role in male reproduction. Mol Hum Reprod.

[20] Yamaguchi M, Mori S (1990). Effect of calcium-binding protein regucalcin on hepatic protein synthesis: inhibition of aminoacyl-tRNA synthetase activity. Mol Cell Biochem.

[21] Yamaguchi M, Nishina N (1995). Characterization of regucalcin effect on proteolytic activity in rat liver cytosol: relation to cysteinyl-proteases. Mol Cell Biochem.

[22] Lowenstein CJ, Dinerman JL, Snyder SH (1994). Nitric oxide: a physiologic messenger. Ann Intern Med.

[23] Liu S, Shia D, Liu G, Chen H, Liu S, Hu Y (2000). Roles of Se and NO in apoptosis of hepatoma cells. Life Sci.

[24] Abou-Elella AM, Siendones E, Padillo J, Montero JL, De la Meta M, Relat JM (2002). Tumour necrosis factor-alpha and nitric oxide mediate apoptosis by d-galactosamine in a primary culture of rat hepatocytes: exacerbation of cell death by cocultured Kupper cells. Can J Gastroenterol.

[25] Izumi T, Tsurusaki Y, Yamaguchi M (2003). Suppressive effect of endogenous regucalcin on nitric oxide synthase activity in cloned rat hepatoma H4-II-E cells overexpressing regucalcin. J Cell Biochem.

[26] Misawa H, Inagaki S, Yamaguchi M (2002). Suppression of cell proliferation and deoxyribonucleic acid synthesis in cloned rat hepatoma H4-II-E cells overexpressing regucalcin. J Cell Biochem.

[27] Belloma G, Perotti M, Taddei F, Mirabelli F, Finardi G, Nicotera P, Orrenius S (1992). Tumor necrosis factor α induces apoptosis in mammary adenocarcinoma cells by an increase in intranuclear free Ca^2+^ concentration and DNA fragmentation. Cancer Res.

[28] Hukkanen M, Hughes FJ, Buttery LD, Gross SS, Evans TJ, Seddon S, Riveros-Moreno V, MacIntyre I, Polak JM (1995). Cytokine stimulated expression of inducible nitric oxide synthase by mouse, rat, and human osteoblast-like cells and its functional role in osteoblast metabolic activity. Endocrinology.

[29] Wink DA, Hanbauer I, Laval F, Cook JA, Krishna MC, Mifchell JB (1994). Nitric oxide protects against the cytotoxic effects of reactive oxygene species. Ann N Y Acad Sci.

[30] Izumi T, Yamaguchi M (2004). Overexpression of regucalcin suppresses cell death in cloned rat hepatoma H4-II-E cells induced by tumor necrosis factor-α or thapsigargin. J Cell Biochem.

[31] Matsuyama S, Kitamura T, Enomoto N, Fujita T, Ishigami A, Handa S, Maruyama N, Zheng D, Ikejima K, Takei Y, Sato N (2004). Senescence marker protein-30 regulates Akt activity and contributes to cell survival in Hep G2 cells. Biochem Biophys Res Commun.

[32] Ishigami A, Fujita T, Handa S, Shirasawa T, Koseki H, Kitamura T, Enomoto N, Sato N, Shimosawa T, Maruyama N (2002). Senescence marker protein-30 knockout mouse liver is highly susceptible to tumor necrosis factor-alpha- and Fas-mediated apoptosis. Am J Pathol.

[33] Alikhani M, Alikhani Z, He H, Liu R, Popek BI, Graves DT (2003). Lipopolysaccharides indirectly stimulate apoptosis and global induction of apoptic genes in fibroblasts. J Biol Chem.

[34] Nolan Y, Vereker E, Lynch AM, Lynch MA (2003). Evidence that lipopolysaccaride-induced cell death is mediated by accumulation of reactive oxygen species and activation of p38 in rat cortex and hippocampus. Exp Neurol.

[35] Izumi T, Yamaguchi M (2004). Overexpression of regucalcin suppresses cell death and apoptosis in cloned rat hepatoma H4-II-E cells induced by lipopolysaccharide, PD98059, dibucaine, or Bay K 8644. J Cell Biochem.

[36] Zelivianshi S, Spellman M, Kellerman M, Kakitelashvilli V, Zhou XW, Lugo E, Lee MS, Taylor R, Daris TL, Hauke R, Lin MF (2003). ERK inhibitor PD 98059 enhances docetaxel-induced apoptosis of androgen-independent human prostate cancer cells. Int J Cancer.

[37] Arita K, Utsumi T, Kato A, Kanno T, Kobuchi H, Inoue B, Akiyama J, Utsumi M (2000). Mechanism of dibucaine-induced apoptosis in promyelocytic leukemia cells (HL-60). Biochem Pharmacol.

[38] Giuliano M, Bellacvia G, Lauricella M, D’Anneo A, Vassallo B, Vento R, Tesoriere G (2004). Staurosporine-induced apoptosis in Chang liver cells is associated with down-regulation of Bcl-2 and Bcl-XL. Int J Mol Med.

[39] Jones DP, McConkey DJ, Nicotera P, Orrenius S (1989). Calcium-activated DNA fragmentation in rat liver nuclei. J Biol Chem.

[40] Farber JL (1981). The role of calcium in cell death. Life Sci.

[41] Yamaguchi M, Sakurai T (1991). Inhibitory effect of calcium-binding protein regucalcin on Ca^2+^-activated DNA fragmentation in rat liver nuclei. FEBS Lett.

[42] Bachs O, Carafolli E (1995). Calmodulin and calmodulin-binding proteins in liver cell nuclei. J Biol Chem.

[43] Omura M, Yamaguchi M (1999). Regulation of protein phosphatase activity by regucalcin localization in rat liver nuclei. J Cell Biochem.

[44] Nicotera P, McConkey DJ, Jones DP, Orrenius S (1989). ATP stimulates Ca^2+^ uptake and increases the free Ca^2+^ concentration in isolated rat liver nuclei. Proc Natl Acad Sci USA.

[45] Christensen SB, Andersen A, Kromann H, Treiman M, Tombal B, Denmeads S, Isaacs JT (1999). Thapsigargin analogues for targeting programmed death of androgen-independent prostate cancer cells. Bioorg Med Chem.

[46] Tombal B, Weeraratna AT, Denmeade SR, Isaacs JT (2000). Thapsigargin induces a calmodulin/calcineurin-dependent apoptotic cascade responsible for the death of prostatic cancer cells. Prostate.

[47] Cohen JJ, Duke RC (1984). Glucocorticoid activation of a calcium-dependent endonuclease in thymocytes nuclei leads to cell death. J Immunol.

[48] Pereira M, Millot J-M, Sebille S, Manfait M (2002). Inhibitory effect of extracellular Mg^2+^ on intracellular Ca^2+^ dynamic changes and thapsigargin-induced apoptosis in human cancer MCF7 cells. Mol Cell Biochem.

[49] Cano-Abad MF, Villarroya M, Garcia AG, Gabilan NH, Lopez MG (2001). Calcium entry through L-type calcium channels causes mitocondrial disruption and chromaffin cell death. J Biol Chem.

[50] Fukaya Y, Yamaguchi M (2005). Overexpression of regucalcin suppresses cell death and apoptosis in cloned rat hepatoma H4-II-E cells induced by insulin or insulin-like growth factor-I. J Cell Biochem.

[51] Spinozzi F, Pagliacci MC, Migliorati G, Moraca R, Grignami F, Riccardi C, Nicoletti I (1994). The natural tyrosine kinase inhibitor genistein produces cell cycle arrest and apoptosis in Jurkat T-leukemia cells. Leuk Res.

[52] Katsumata T, Yamaguchi M (1998). Inhibitory effect of calcium-binding protein regucalcin on protein kinase activity in the nuclei of regenerating rat liver. J Cell Biochem.

[53] Hunter T (1995). Protein kinases and phosphatases: the Yin and Yang of protein phosphorylation and signaling. Cell.

[54] Omura M, Yamaguchi M (1999). Enhancement of neutral phosphatase activity in the cytosol and nuclei of regenerating rat liver: role of endogenous regucalcin. J Cell Biochem.

[55] Gamet-Payrastre L, Li P, Lumeau S, Cassar G, Duport NA, Chevolleau S, Gasc N, Tulliez J, Terce F (2000). Sulforaphane, a naturally occurring isothiocyanate, induces cell cycle arrest and apoptosis in HT29 human colon cancer cells. Cancer Res.

[56] Fimognari C, Nusse M, Cesari R, Iori R, Cantelli-Forti G, Hrelia P (2002). Growth inhibition, cell-cycle arrest and apoptosis in human T-cell leukemia by the isothiocyanate sulforaphane. Carcinogenesis.

[57] Singh AV, Xiao D, Lew KL, Dhir R, Singh SV (2004). Sulforaphane induces caspase-mediated apoptosis in cultured PC-3 human prostate cancer cells and retards growth of PC-3 xenografts in vivo. Carcinogenesis.

[58] Fukaya Y, Yamaguchi M (2005). Overexpression of regucalcin suppresses apoptotic cell death in the cloned rat hepatoma H4-II-E cells induced by a naturally occurring isothiocyanate sulforaphane. Int J Mol Med.

[59] Nakagawa T, Yamaguchi M (2005). Hormonal regulation on regucalcin mRNA expression in cloned normal rat kidney proximal tubular epithelial NRK52E cells. J Cell Biochem.

[60] Nakagawa T, Yamaguchi M (2008). Nuclear localization of regucalcin is enhanced in culture with protein kinase C activation in cloned normal rat kidney proximal tubular epithelial NRK52E cells. Int J Mol Med.

[61] Nakagawa T, Sawada N, Yamaguchi M (2005). Overexpression of regucalcin suppresses cell proliferation of cloned normal rat kidney proximal tubular epithelial NRK52E cells. Int J Mol Med.

[62] Nakagawa T, Yamaguchi M (2005). Overexpression of regucalcin suppresses apoptotic cell death in cloned normal rat kidney proximal tubular epithelial NRK52E cells: change in apoptosis-related gene expression. J Cell Biochem.

[63] Nakagawa T, Yamaguchi M (2007). Overexpression of regucalcin suppresses cell response for tumor necrosis factor-α or transforming growth factor-β1 in cloned normal rat kidney proximal tubular epithelial NRK52E cells. J Cell Biochem.

[64] Vogelstein B, Lane D, Levine AJ (2000). Surfing the p53 network. Nature.

[65] Zou H, Hanzel WJ, Liu X, Lutschg A, Wang X (1997). Apaf-l, a human protein homologous to C. elegans CED-4, participates in cytochrome c-dependent activation of caspase-3. Cell.

[66] Widmann C, Gibson S, Johnson GL (1988). Caspase-dependent cleavage of signaling proteins during apoptosis. A turn-off mechanism for anti-apoptotic signals. J Biol Chem.

[67] Dieguez-Acuna FJ, Polk WW, Ellis ME, Simmonds PL, Kushleika JV, Woods JS (2004). Nuclear factor kappaB activity determines the sensitivity of kidney epithelial cells to apoptosis: implications for mercury-induced renal failure. Toxicol Sci.

[68] Yamaguchi M (2013) Suppressive role of regucalcin in liver cell proliferation: Involvement in carcinogenesis. Cell Prolif (in press) 10.1111/cpr.12036 PMC6496855 23692083

